# Gabriel Audebert's polychrome busts

**DOI:** 10.1017/S2045796022000415

**Published:** 2022-09-09

**Authors:** Marc Décimo

**Affiliations:** University of Paris Ouest Nanterre, Paris, France

Sculptor? Painter? This former hairdresser opened *his* museum in Pleaux (Cantal, France) in an old bistro owned by his parents, who also used to be farmers. His vacation home was located just across, in Place d'Empeyssine, also known as Place du Champ-de-foire. There he set up the busts he made all the winters he spent in Ormesson-sur-Marne, from his retirement in 1986 until his death.

A quick survey would place the size of the collection at roughly 250 busts.

Whilst it involved soaking newspapers he had either carefully preserved or were brought to him from elsewhere, the technique he employed, he clarified, surely was not *papier mâché*, but a technique he had invented himself.

Once the sheets of newspaper had disintegrated to the point of liquefaction, he would, armed with a drill, grind the resulting mass before carefully mixing in some amount of glue in order to make it denser. This process of pressing and compressing transfigured the mass into something that was less paper and more camembert, which he then put in an oven to dry. One batch could make twenty to thirty truckles at a time.

But the labour did not stop there. Once again, the paper blocks had to be ground and pulverised, chipped and sifted, and then further agglomerated with glue until another mass was obtained. Only then could the paste be transferred to a framework, which consisted of a rod and a wire mesh, shaped and finally sculpted. Audebert had to use sharp tools, the carpenter's or the dentist's. A scalpel perhaps? Applying colour was for him the easiest part of the process. After all, didn't he use to dye hair?

**Fig. 1.**
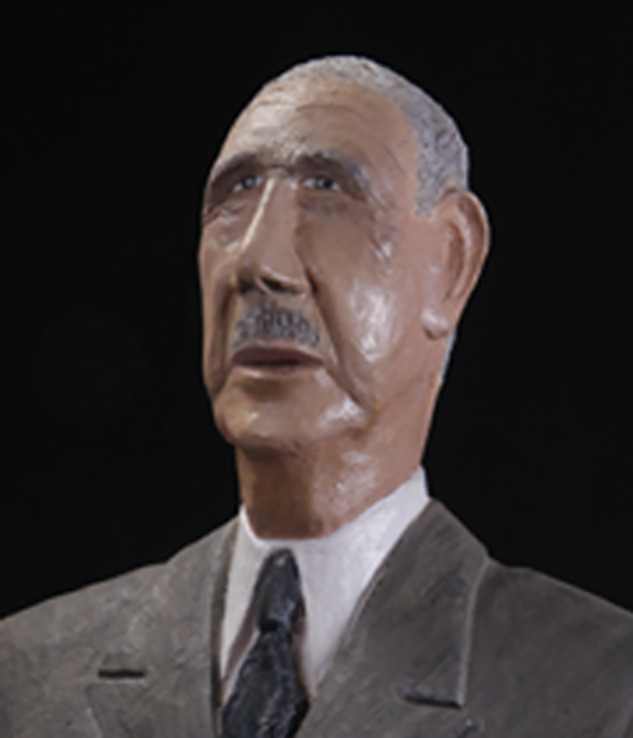
*General de Gaulle,* before 1989 (65.4 × 50.6 × 20.7 cm), Green Cloud, Argentat-sur-Dordogne. Courtesy Yves Desbuquois and Green Cloud Museum Dordogne Valley.

He made masks (dyes allowed him to imitate bronze), and then caricatures of politicians, for example. One might recognise, among many others, General de Gaulle [Fig. 1], François Mitterrand, Michel Rocard, Valéry Giscard d'Estaing, Jacques Chirac, all well-known figures from the 1960s to the 2000s.

**Fig. 2.**
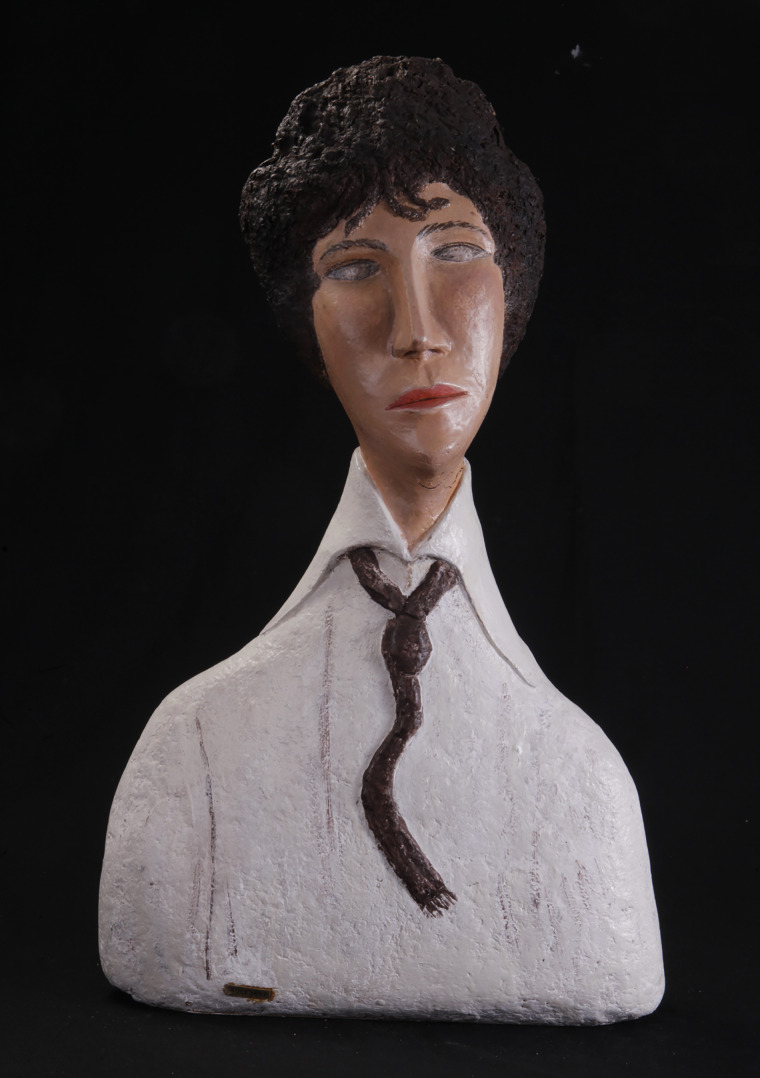
Gabriel Audebert, according to Amedeo Modigliani, *Lady with Tie* (68.5 × 40.8 × 21 cm), Green Cloud, Argentat-sur-Dordogne. Courtesy Yves Desbuquois and Green Cloud Museum Dordogne Valley.

Then came the period inspired by paintings: Van Gogh, Modigliani [Fig. 2], Toulouse-Lautrec, and so many others.

Towards the end of his life, he devoted himself to creating an inventory of the crosses of Cantal. Before reproducing them, always according to the same technique, he photographed and documented them. Dyes allowed him to imitate stone. He was always amused whenever a visitor weighed his sculptures and found them to be rather light.

This quest of his is also the subject of an illustrated book seventy-nine pages thick, published in 1995, and which he paid for himself: *La nouvelle sculpture, les croix du Massif central*, Ormesson-sur-Marne (24 rue des deux-communes, 94490), Gabriel Audebert. Its printing was entrusted to the Gerbert publishing house in Aurillac.

Gabriel Audebert was born in Pleaux on August 29, 1924. He died in Brunoy (Essonne) on March 29, 2007. Having opened a salon in Paris first at 22 Rue de la Huchette, then another at 88 Boulevard Saint-Germain, his career as a hairdresser began in 1950. He sometimes held exhibits in Ormesson-sur-Marne and in Pleaux (places he lived in), and several times in Paris at the Palais des Congrès during the Mondial Coiffure Beauté. He also photographed and made postcards of some of his works. Now the busts are kept in Argentat-sur-Dordogne (Green Cloud Museum Dordogne Valley) and the crosses in Pleaux (The Association of Friends Xaintrie cantalienne).
